# Skin microbiota analysis-inspired development of novel anti-infectives

**DOI:** 10.1186/s40168-020-00866-1

**Published:** 2020-06-05

**Authors:** Yao Liu, Yuanzhen Liu, Zixiu Du, Lidan Zhang, Juan Chen, Zhen Shen, Qian Liu, Juanxiu Qin, Huiying Lv, Hua Wang, Lei He, Junlan Liu, Qian Huang, Yuhui Sun, Michael Otto, Min Li

**Affiliations:** 1 Department of Laboratory Medicine, Renji Hospital, School of Medicine, Shanghai Jiaotong University, Shanghai, 200127 China; 2 Key Laboratory of Combinatorial Biosynthesis and Drug Discovery, Ministry of Education, Wuhan University School of Pharmaceutical Sciences, Wuhan, 430071 China; 3 School of Pharmacy, Shanghai Jiaotong University, Shanghai, 200240 China; 4 Pathogen Molecular Genetics Section, Laboratory of Bacteriology, National Institute of Allergy and Infectious Diseases, U.S. National Institutes of Health, Bethesda, MD 20814 USA

## Abstract

**Background:**

The alarming spread of antimicrobial resistance requires the development of novel anti-infective drugs. Despite the recent research focus on the human microbiome and its likely value to understand and exploit inter-bacterial inhibitory phenomena as a source for antimicrobial strategies, the human microbiota has barely been investigated for the purpose of drug development.

**Results:**

We performed a large screen analyzing over 3000 human skin isolates to evaluate bacterial competition within the human skin microbiota as a basis for the development of anti-infective therapeutics. We discovered a *Staphylococcus hominis* strain with strong and broad activity against Gram-positive pathogens that was mediated by the bacteriocin micrococcin P1 (MP1). In “probiotic” approaches, this strain led to reduced *Staphylococcus aureus* infection and accelerated closure of *S. aureus*-infected wounds. Furthermore, we used a nanoparticle strategy to overcome the physico-chemical limitations often encountered with natural substances such as MP1 and demonstrate a significant reduction of *S. aureus* infection by MP1-loaded nanoparticles.

**Conclusions:**

Our study gives examples of how analysis of bacterial interactions in the human microbiota can be explored for the development of novel, effective anti-infective strategies.

Video Abstract

## Background

Since the discovery of penicillin, the majority of antibiotics in clinical use are unmodified or modified derivatives of natural products that are produced by microorganisms to kill competing bacteria and were originally isolated from environmental sources. Nowadays, the situation of increasing antimicrobial resistance in many bacterial pathogens calls for novel strategies to detect and develop new antibiotics or alternatives to conventional antibiotics [1]. While relationships within the human microbiota have gained much recent interest and it is believed that members of the human microbiota produce substances to control the overgrowth of pathogenic competitors [2], few studies have used analysis of competitive phenomena among the human microbiota for the purpose of drug discovery and development.

The skin represents one of several epithelia on the human body where microbial communities exist and live in constant interaction with the host and each other. Commensal skin bacteria are believed to be fundamental for the maintenance of the skin barrier and have been implicated in the protection from infection by pathogenic bacteria and other microorganisms [3]. Furthermore, dysbiosis of the human skin microbiome has been associated with diseases such as psoriasis, atopic dermatitis, and acne [4]. It is believed that skin commensals exert their protective function in many ways. In addition to educating and supporting the host immune system [5–7], they may produce substances to directly impair the growth or virulence of competitors [8, 9].

Staphylococci represent some of the most frequent skin commensals in humans. In contrast to the infamously pathogenic and multidrug-resistant *Staphylococcus aureus* [10], most other staphylococci—almost all of which are categorized as coagulase-negative staphylococci (CoNS)—only cause infections in compromised hosts or are genuinely non-pathogenic [11]. Several findings support the notion that CoNS represent a treasure trove for the development of novel anti-infectives. For example, a lack of bacteriocin-producing CoNS has been associated with increased colonization by *S. aureus* on the skin of subjects with atopic dermatitis [9]. Furthermore, lugdunin, a novel antibiotic produced by a human nasal *Staphylococcus lugdunensis* isolate*,* has a strong inhibitory capacity toward *S. aureus* and a range of other bacteria [12]. Finally, inhibition of quorum-sensing virulence regulation in *S. aureus* by cross-inhibitory signals from CoNS may be exploited to control the virulence of *S. aureus* [8, 13]. Nevertheless, despite these recent findings, efforts to translate competitive potencies of skin commensals, such as CoNS, into potential anti-infective therapeutics are still in their infancy.

In the present study, we analyzed the skin microbiota in a large cohort of children, young adults, and seniors for bacterial competition phenomena. We found that the species *Staphylococcus hominis* frequently produces antimicrobial substances and focused on the substance, micrococcin P (MP1), produced by a strain of *S. hominis* that showed the strongest and broadest antimicrobial activity. We demonstrate the potential of the *S. hominis* producer in “probiotic” types of topical application to reduce infection and accelerate healing of infected wounds; while using a nanoparticle strategy, we developed an MP1 formula that significantly reduced *S. aureus* local and systemic infection. By deriving two specific potential ways to control infection by *S. aureus* and other Gram-positive pathogens from the study of the human microbiota, our study underlines the potential of human microbiota analysis-inspired strategies for anti-infective drug development.

## Results

### Analysis of competition among skin bacteria and selection of highly active *S*. *hominis* isolate

To serve as a basis for the development of an efficient anti-infective, we performed a large screen of the human skin microbiota that was aimed to discover a strongly and broadly competitive commensal skin isolate. While nowadays, DNA sequencing-based analysis of the human microbiota has become increasingly popular, which in addition to cataloging purposes and correlation of dysbiosis with diseases has also led to the identification of genetic systems encoding previously unknown antimicrobial substances [14], we here performed a culturing-based approach. First, because this approach to our knowledge has not previously been performed to that end on a similarly large scale, and second as we reasoned that bacteria need to be culturable for the straightforward analysis and production of antimicrobial substances, or to be used in a live bacteria-based “probiotic” type of regimen.

**Fig. 1:**
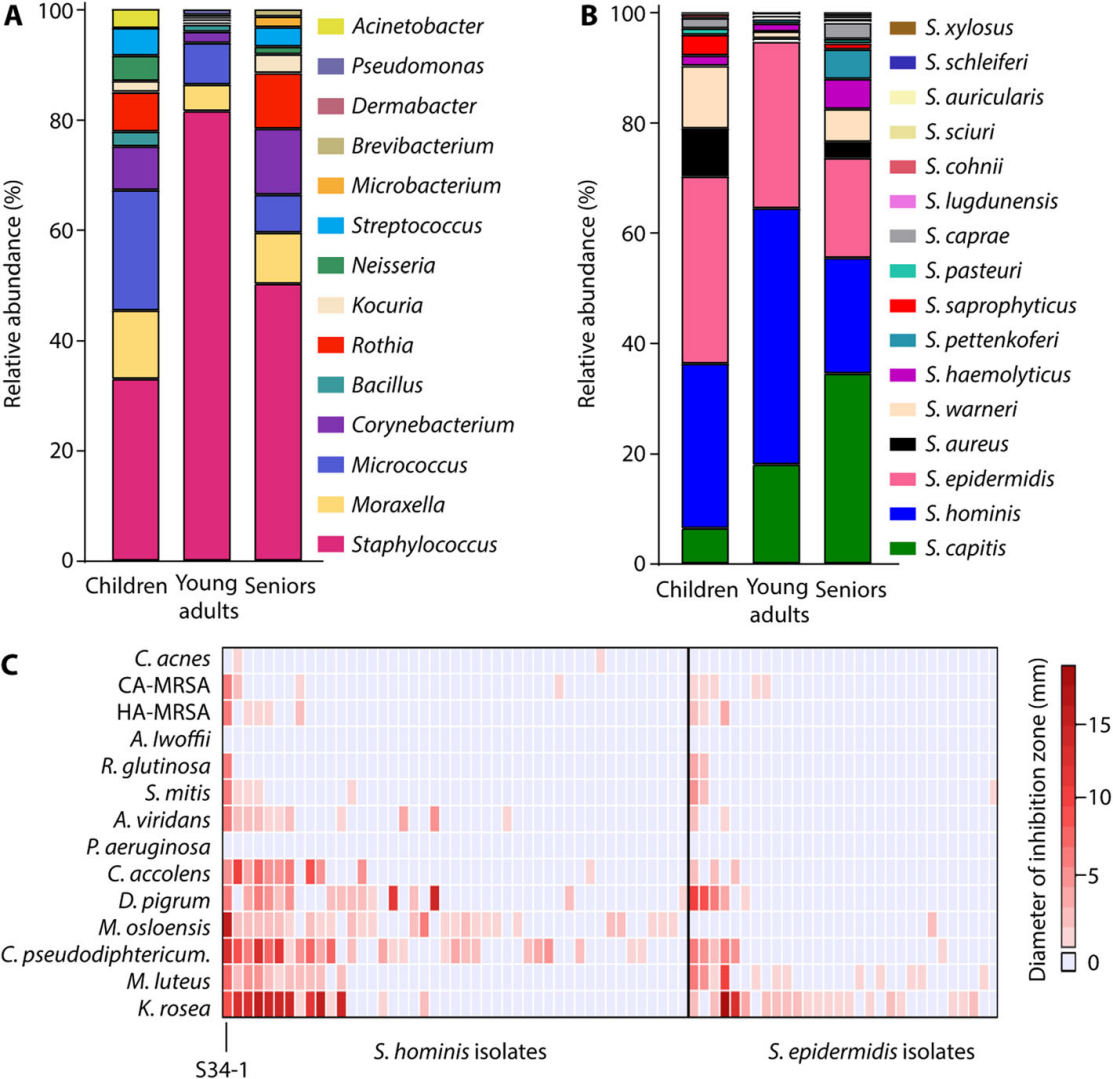
Antimicrobial activities among main constituents of the human skin microbiota. **a** Relative abundance of main genera among culturable bacteria from the skin of human volunteers in different age groups. **b** Relative abundance of staphylococcal species. **c** Antimicrobial activities against a series of bacteria of randomly selected *S. hominis* and *S. epidermidis* isolates by agar disk diffusion assay. Two μl of bacterial suspensions were spotted on the agar surface. The data of the selected strain S34-1 is marked

**Fig. 2:**
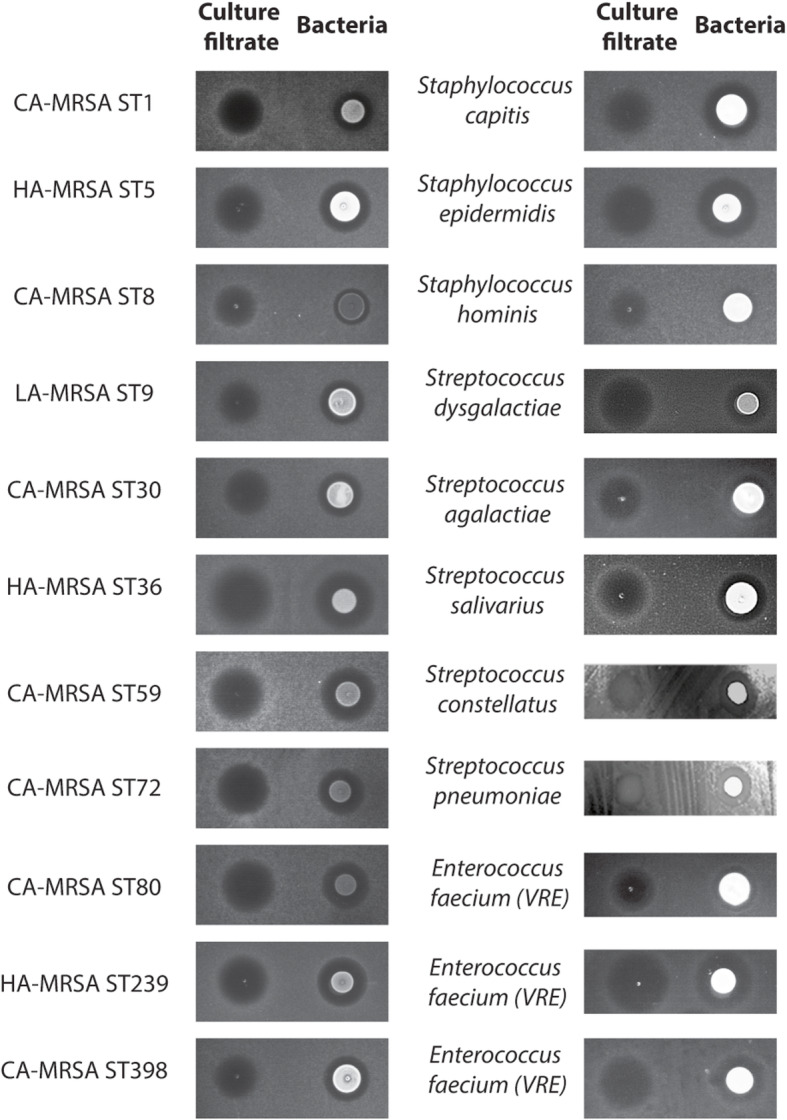
Activity of *S. hominis* S34-1 and S34-1 culture filtrate toward Gram-positive pathogens. Culture filtrate or bacteria of *S. hominis* S34-1 were spotted on plates containing the indicated bacterial strains. Refer to “Methods” for strain-specific plate preparation and incubation times

We investigated a total of 3167 bacterial isolates obtained from the cubital fossae of 156 children, 210 young adults, and 160 seniors (Additional Table 1). The genus *Staphylococcus* was the most abundant (31.47%, 80.23%, and 46.97%, in children, young adults, and seniors, respectively) (Fig. 1a). The difference of staphylococcal abundance in the adult versus the other groups was statistically significant (children vs. young adults, young adults vs. seniors, both *p* < 0.0001, chi-square test). Overall, the species *Staphylococcus hominis*, *S. epidermidis*, and *S. capitis* dominated at 33.83%, 26.10%, and 20.18%, respectively, among the obtained *Staphylococcus* isolates (Fig. 1b). There was a significantly lower number of the pathogenic species *S. aureus* as compared to all other staphylococci (CoNS) in the young adult group (children vs. young adults, *p* < 0.0001, young adults vs. seniors, *p* < 0.0001, chi-square test). From the most frequently found *S. hominis* and *S. epidermidis*, we randomly selected 112 non-duplicate isolates. These isolates were tested for inhibitory activities toward 14 strains of bacteria, most of which are opportunistic skin pathogens (Table 1). We focused on isolates obtained from young adults, as in that group abundance of the pathogenic *S. aureus* was lowest, suggesting pronounced anti-pathogenic activity. We discovered frequent antimicrobial capacities particularly among *S. hominis* isolates (Fig. 1c). One isolate, S34-1, showed especially broad activity, including against methicillin-resistant *S. aureus* (MRSA) and other Gram-positive bacteria. Further testing of this strain and its culture filtrate revealed that it secretes an antimicrobial activity that inhibits the growth of a series of community-, hospital-, and livestock-associated MRSA strains, as well as other multidrug-resistant Gram-positive pathogens, including pathogenic and penicillin-resistant streptococci, vancomycin-resistant enterococci (VRE), and methicillin-resistant CoNS (Table 2, Fig. 2).

**Table 1:** Indicator strains used for screening *S. hominis* and *S. epidermidis* isolates generating antimicrobial substances

Strain	Source	Atmosphere	Growth media
*Corynebacterium pseudodiphtheriticum*	From skin; this study	Aerobic	Sheep blood agar
*Moraxella osloensis*	From skin; this study	Aerobic	Sheep blood agar
*Dolosigranulum pigrum*	From skin; this study	Aerobic	Sheep blood agar
*Corynebacterium accolens*	From skin; this study	Aerobic	Sheep blood agar
*Streptococcus mitis*	From skin; this study	Aerobic	Sheep blood agar
*Rosella glutinosa*	From skin; this study	Aerobic	Sheep blood agar
*Micrococcus luteus*	From skin; this study	Aerobic	TSA
*Aerococcus viridans*	From skin; this study	Aerobic	Sheep blood agar
*Kocuria rosea*	From skin; this study	Aerobic	Sheep blood agar
*Pseudomonas aeruginosa*	From skin; this study	Aerobic	TSA
*Acinetobacter Iwoffii*	From skin; this study	Aerobic	TSA
*Cutibacterium acnes*	From acne; this study	Anaerobic	Sheep blood agar
CA-MRSA (ST59) Strain RJ-2	[15]	Aerobic	TSA
HA-MRSA (ST5) Strain HS72	[16]	Aerobic	TSA

**Table 2:** Multidrug-resistant Gram-positive pathogens tested for sensitivity toward *S. hominis* S34-1 activity

Type of species	Comment	Reference	Antibiograms^a^
CA-MRSA^a^ (ST1)	Strain MW2 (USA400)	[17]	OXA, CFZ, PEN, CXM, FOX
HA-MRSA (ST5)	Strain HS72, pathogenic, isolated from abscess	[16]	OXA, LVX, CLI, CFZ, FOS, ERY, AMK, PEN, GEN, FOX
CA-MRSA (ST8)	SF8300 (USA300)	[18]	OXA, CFZ, ERY, PEN, CXM, FOX
LA-MRSA (ST9)	Pathogenic, isolated from bovine mastitis	[19]	OXA, LVX, CLI, CFZ, ERY, TGC, PEN, CXM, FOX
CA-MRSA (ST30)	Strain SF-1497 (USA1100)	[18]	OXA, CFZ, PEN, CXM, FOX
HA-MRSA (ST36)	Strain SF 1208 (USA200)	[18]	OXA, LVX, CLI, CFZ, ERY, SAM, PEN, CXM, FOX
CA-MRSA (ST59)	Strain RJ-2, pathogenic, isolated from skin infection	[15]	OXA, CLI, ERY, PEN, CXM, FOX
CA-MRSA (ST72)	Strain CN1, South Korea	[20]	OXA, CFZ, ERY, PEN, CXM, FOX
CA-MRSA (ST80)	Strain 07-02662, Germany	[21]	OXA, ERY, PEN, FOX
HA-MRSA (ST239)	Strain HS770, isolated from sputum	[16]	OXA, LVX, CLI, CFZ, ERY, AMK, SAM
CA-MRSA (ST398)	Pathogenic, isolated from blood	[22]	OXA, ERY, PEN, FOX
*Staphylococcus capitis*	Pathogenic, isolated from pleural fluid	This study	OXA, PEN, CIP, SXT, MXF, LVX
*Staphylococcus epidermidis*	Pathogenic, isolated from blood	This study	OXA, PEN, CIP, ERY, MXF, LVX
*Staphylococcus hominis*	Pathogenic, isolated from blood	This study	OXA, PEN, ERY
*Streptococcus dysgalactiae*	Pathogenic, isolated from sputum	This study	ERY
*Streptococcus agalactiae*	Pathogenic, isolated from urine	This study	CLI, LVX, CIP, MXF
*Streptococcus salivarius*	Pathogenic, isolated from blood	This study	PEN
*Streptococcus constellatus*	Pathogenic, isolated from blood	This study	PEN
*Streptococcus pneumoniae*	Pathogenic, isolated from sputum	This study	PEN, CLI, OXA, LVX, ERY, CLR
VRE (*Enterococcus faecium*)	Pathogenic, isolated from catheter	This study	VAN, PEN, CIP, LVX, ERY, LVX, TEC, TET, AMP, MXF, GEN, CLI, FOF
VRE (*Enterococcus faecium*)	Pathogenic, isolated from urine	This study	VAN, PEN, AMP, CIP, LVX, MXF, ERY, CLI
VRE (*Enterococcus faecium*)	Pathogenic, isolated from urine	This study	VAN, PEN, CIP, LVX, ERY, LVX, TEC, AMP, MXF, STR, CLI, FOF

^a^Antibiograms were determined by disc diffusion on Mueller-Hinton agar according to Clinical and Laboratory Standards Institute guidelines

*Abbreviations*: OXA oxacillin, PEN penicillin, LVX levofloxacin, CFZ cephazolin, CXM cefuroxime, FOX cefoxitin, CLI cephalexin, FOS fosfomycin, ERY erythromycin, AMK amikacin, GEN gentamicin, TGC tigecycline, SAM sulbactam/ampicillin, SXT sulphamethoxazole/trimethoprim, MXF moxifloxacin, CIP ciprofloxacin, TET tetracycline, CLR clarithromycin, VAN vancomycin, TEC teicoplanin, STR streptomycin, FOF nitrofurantoin, LA live-associated, CA community-associated, HA hospital-associated

### Identification of the broad-spectrum antimicrobial substance produced by the active *S. hominis* isolate

**Fig. 3:**
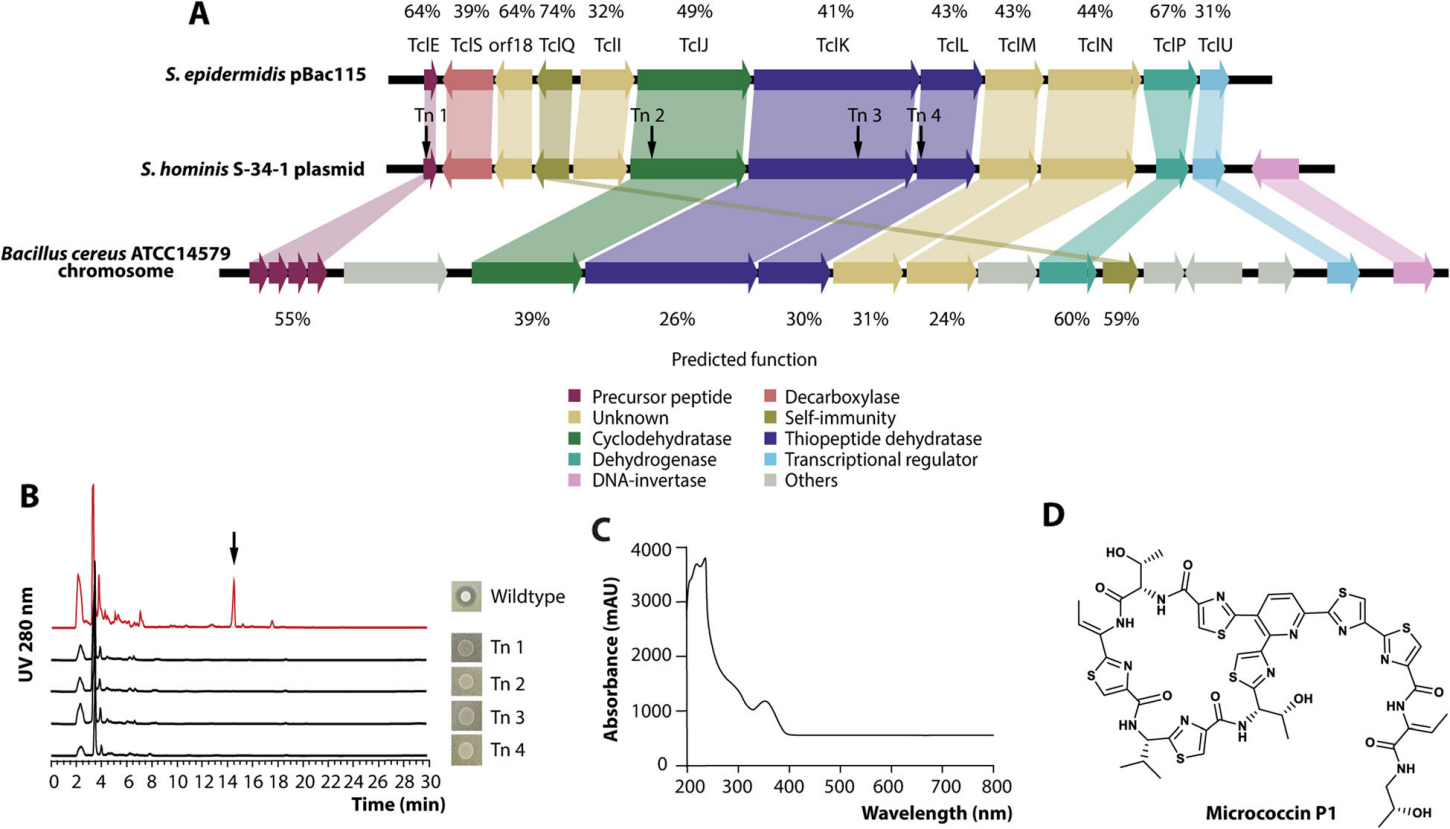
Identification of the antimicrobial activity produced by *S. hominis* S34-1. **a** Genetic locus producing the antimicrobial activity and alignment with homologous loci found on plasmid pBac115 of an *S. epidermidis* strain and on the chromosome of *B. cereus* ATCC14579, described to produce the thiopeptide micrococcin P1. Amino acid similarity of encoded protein products is shown as well as putative annotation of protein function. **b** RP-HPLC chromatography of culture filtrates of S34-1 wild-type and the transposon mutant strains that lacked activity. **c** UV spectrum of purified MP1. **d** Structure of micrococcin P1

To identify the nature of the antimicrobial substance produced by *S. hominis* S34-1, we used a two-pronged biochemical and genetic approach. First, to identify the genes responsible for the antimicrobial activity, we genome-sequenced the S34-1 isolate and performed mariner-transposon-based mutagenesis with a plasmid, we previously had developed for use in staphylococci [23]. We built a transposon library of 21,000 clones that were screened for the disappearance of inhibition on lawns of a sensitive MRSA strain. Absence of inhibition was observed in four clones, in which the transposon insertions mapped to four open reading frames (ORFs) all found in close vicinity on a 29,577 basepair plasmid. These as well as the surrounding ORFs showed strong homology to micrococcin P1 (MP1) biosynthesis clusters first described in *Bacillus cereus* [24] and recently also in a *Staphylococcus epidermidis* strain [25]. In the latter, they are also encoded on a plasmid, which is similar in size to the ~29.6-kb plasmid of *S. hominis* S34-1 (Fig. 3a).

**Fig. 4:**
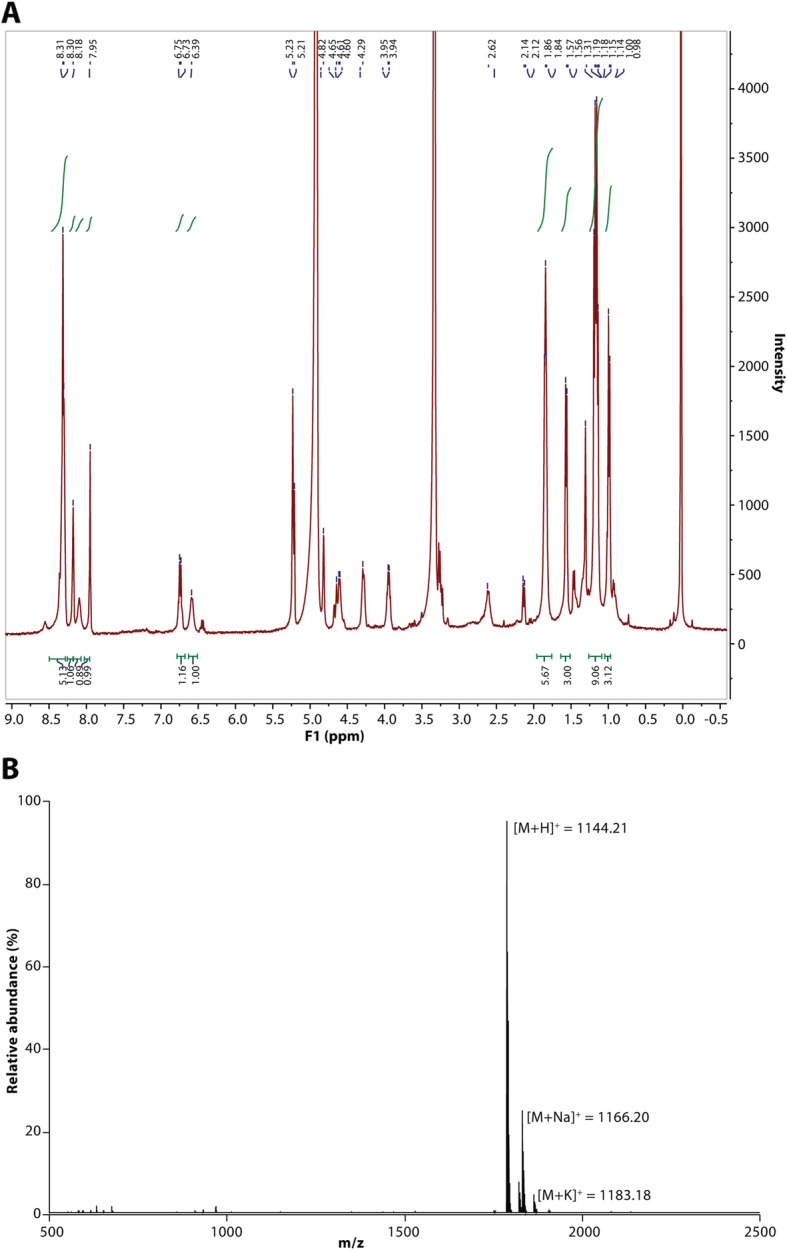
1D-NMR and ESI-MS spectra of purified MP1. **a** 1D-^1^H-NMR. **b** ESI-MS.

MP1 is a member of the thiopeptide family of bacterial peptides, which are 12- to 17-amino-acid in length and heavily post-translationally modified. They all contain sulfur-containing heterocyclic rings and dehydrated residues within a macrocyclic structure [26]. The amino acid sequence of the part of the precursor peptide-encoding gene that gives rise to mature MP1 was the same in *S. hominis* S34-1 and the *S. epidermidis* strain, in which MP1 production was previously detected [25]. This strongly suggested that S34-1 also produces MP1. Reversed-phase high-pressure liquid chromatography (HPLC) revealed differences between extracts from S34-1 wild-type and the four transposon mutants in only one major signal (Fig. 3b), which showed a UV spectrum characteristic of a post-translationally modified heterocyclic peptide (Fig. 3c). This substance, which had antimicrobial activity, was purified and using high-pressure chromatography/electrospray ionization mass spectrometry (HPLC/ESI-MS) and nuclear magnetic resonance (NMR) measurements were identified as MP1 (Fig. 3d, Fig. 4a and b).

### Analysis of “probiotic” therapy potential of MP1-producing *S. hominis*

**Fig. 5:**
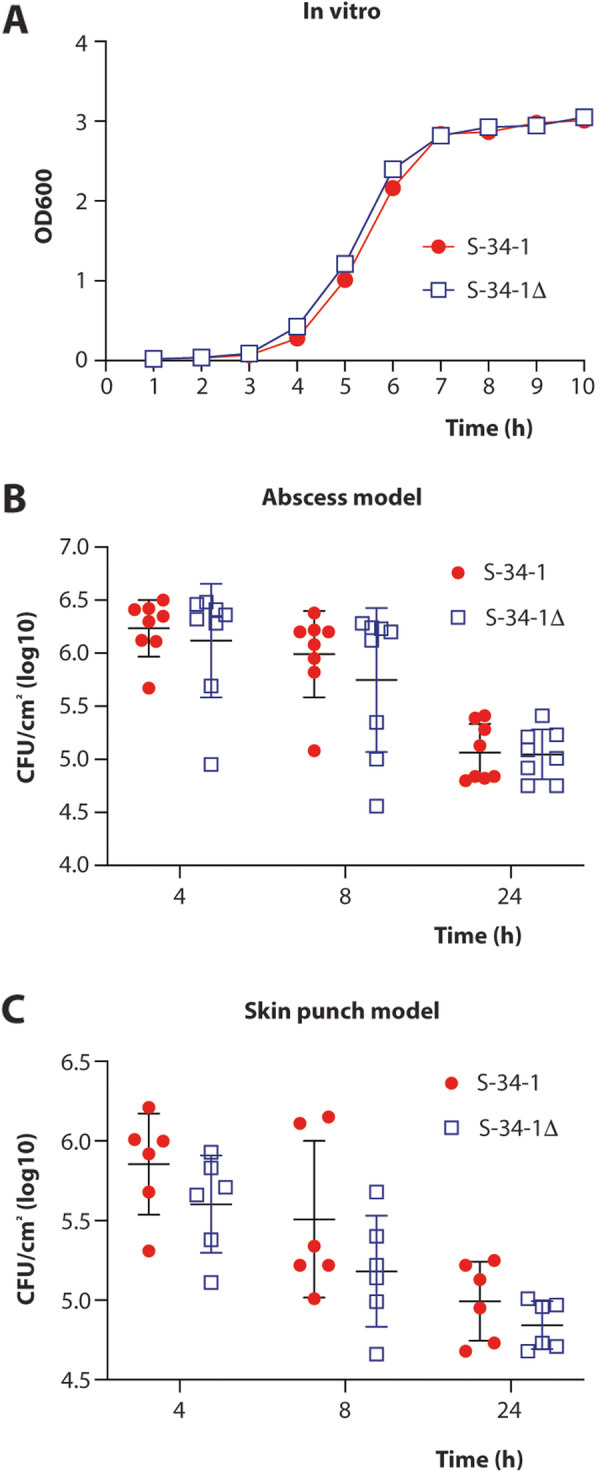
**a In vitro and in vivo growth of*S. hominis*S34-1 and*S. hominis*S34-1Δ.** Data points are averaged from triplicate measurements. **b***S. hominis* CFU in the abscess model (see **Fig.****6**). ***n*** = 8/group and time point. **c***S. hominis* CFU in the skin punch model (see **Fig.****7**). ***n*** = 6/group and time point. **b, c** Statistical analysis was by unpaired ***t*** tests. Differences were not significant (***p***>0.05). Error bars show the mean **±** SD

In addition to conventional anti-infective therapy using antimicrobial pure substances, there is much recent discussion of “probiotic” approaches using live, beneficial bacteria to stimulate immune responses or directly outcompete pathogens [27]. In “probiotic” dietary supplements or fecal transplants for anti-clostridial therapy, such approaches are already in use [28], while for skin diseases they have so far only been proposed [9]. Furthermore, our screen of human isolates was performed based on the assumption that a promising antibacterial producer strain should show pronounced competitive activity against pathogens in vivo. Therefore, to evaluate the potential of *S. hominis* S34-1 as a probiotic-type treatment and validate the results from our screen, we tested the competitive efficiency of *S. hominis* S34-1 in animal models of infection with virulent *S. aureus* as competing pathogen. To be able to pinpoint phenotypes directly to MP1, we compared to one of the *S. hominis* S34-1 transposon mutants in the MP1 biosynthetic cluster that is devoid of MP1 production (named S34-1Δ). This specific mutant was chosen among the four mutants devoid of antimicrobial activity because the insertion was in the structural gene of the peptide, whereas in the others, the insertions were in modification genes. Notably, there was no difference in in vitro or in vivo growth in the used experimental setups between *S. hominis* S34-1 and S34-1Δ, and thus, the phenotypes described in the following are not due to inherently different growth characteristics of those strains (Fig. 5). Furthermore, RNA-Seq analysis of the S34-1Δ mutants strain in comparison to the wild-type strain showed only minor expression differences in mostly metabolic genes, as commonly found as technical variations when comparing isogenic strains. Some of these minor changes may also be interpreted as a slight metabolic adaptation to the production of a bacteriocin but given their nature and low extent, they are extremely unlikely to account for any of the interbacterial interaction phenotypes described in the following (Additional Table 2).

**Fig. 6:**
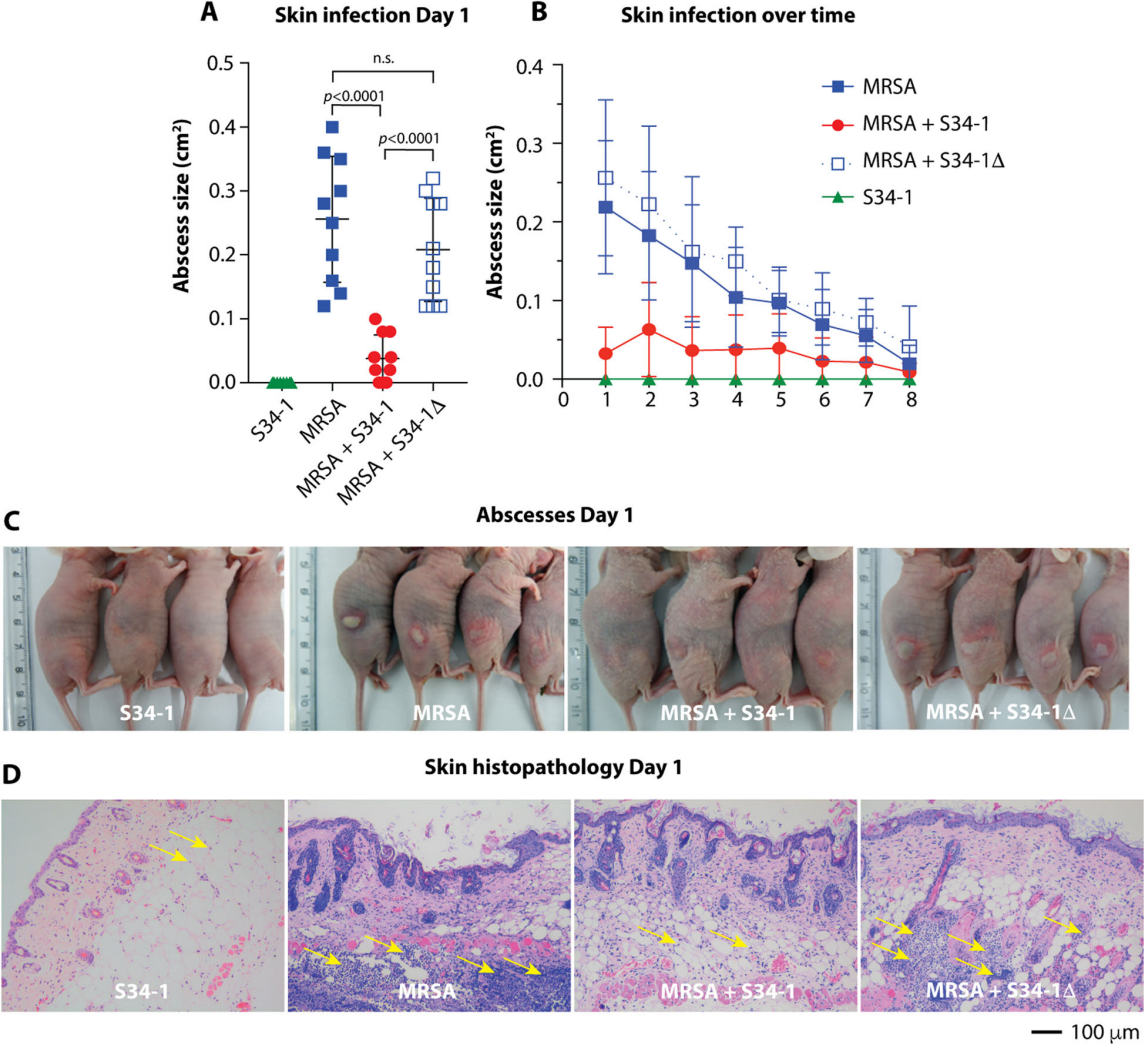
MP1-dependent competitive capacity of *S. hominis* S34-1 against MRSA during skin infection. Mice received 1 × 10^7^ CFU of the indicated bacteria in 100 μl PBS by subcutaneous infection in the right and left flanks at the dorsum. *n* = 5 mice/group. **a** Abscess size comparisons on day 1. Statistical analysis is by 1-way ANOVA with Tukey’s post-test. **b** Abscess sizes over time. **c** Pictures of abscesses on day 1. **d** Skin histopathology on day 1. Note extensive infiltration of granulocytes (yellow arrows) in MRSA and MRSA + S34-1Δ groups. **a, b** Error bars show the mean ± SD. See Fig. 5 for a control experiment comparing CFU of *S. hominis* S34-1 and S-341Δ

**Fig. 7:**
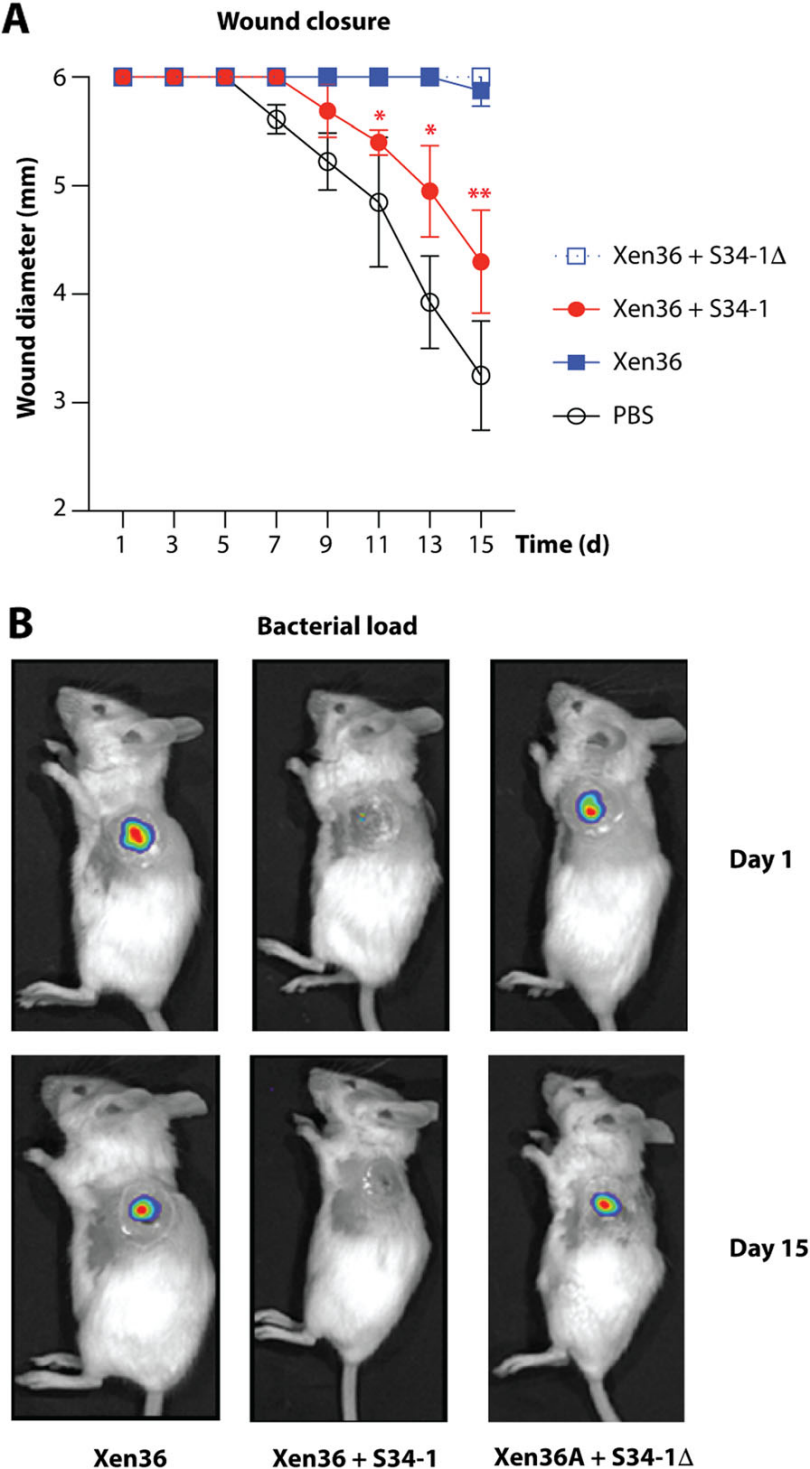
“Probiotic” application of *S. hominis* S34-1 against *S. aureus* wound infection. **a** Wound closure rate. Wound closure is expressed as a decrease in wound size. Mice (*n* = 4/group) received 1 × 10^7^ CFU (in mixtures, 1 × 10^7^ CFU each) of the indicated bacteria, or PBS as control, in wounds created by excision of a round area with a 6-mm diameter. The *S. aureus* strain Xen 36 was used. Error bars show the mean ± SD. **b** Bacterial load in wounds at days 1 and 15 after infection by in vivo imaging of the luminescence expressed by *S. aureus* strain Xen36

**Fig. 8:**
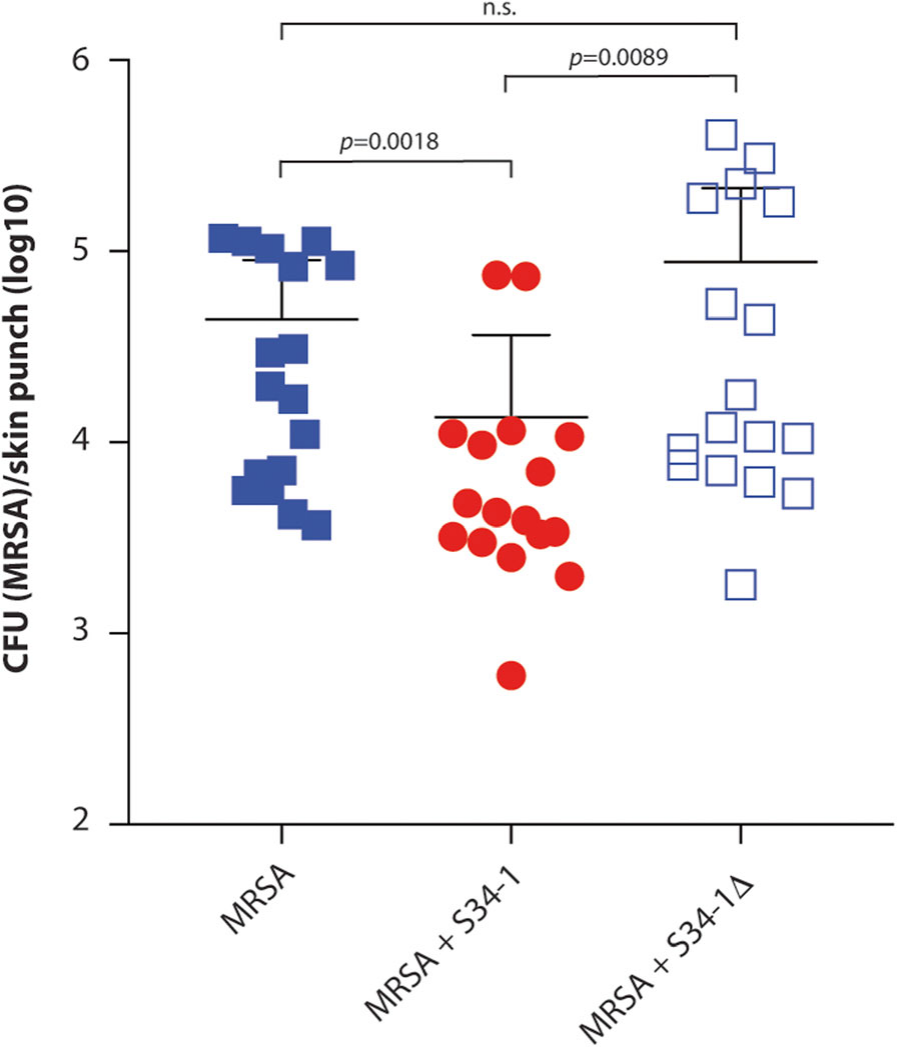
MP1-dependent competitive capacity of *S. hominis* S34-1 against MRSA in a skin punch model. Mice (*n* = 17/group) received 1 × 10^7^ CFU of the indicated bacteria (in mixtures, 1 × 10^7^ CFU each) of the indicated bacteria in a 5-mm square hole cut into a 1-cm diameter square silicone sheet pasted on a shaved area on the posterior lower back. CFU was determined by plating of the dissected colonized skin tissue after 24 h. Statistical analysis is by Kruskal-Wallis with Dunn’s post-test. Error bars show the mean ± SD. See Fig. 5 for a control experiment comparing CFU of *S. hominis* S34-1 and S34-1Δ

Subcutaneous skin infections such as abscesses are among the most frequent disease types associated with *S. aureus* [10]. In a subcutaneous skin infection mouse model, S34-1, but not S34-1Δ, reduced sizes of abscesses caused by a virulent MRSA strain (Fig. 6a–c) and reduced inflammatory phenomena such as granulocyte infiltration (Fig. 6d). Additionally, *S. aureus* is a frequent and dangerous culprit in the exacerbation of chronic wound infection [29]. Co-application of S34-1 with a virulent *S. aureus* strain led to a strong and significant increase of wound healing as compared to the group of mice that received *S. aureus* alone, while co-application with S34-1Δ had no effect (Fig. 7a). Remarkably, the rate of wound healing with the S34-1 therapy of an *S. aureus*-infected wound almost achieved the rate observed in a wound without infection (PBS control group). Furthermore, the application of S34-1 (but not S34-1Δ) led to the eradication of *S. aureus* to non-detectable levels already at day 1 post-application, which persisted throughout the course of the experiment (Fig. 7b). Finally, in a skin-punch mouse model, S34-1 but not S34-1Δ showed a significant capacity to outcompete a virulent MRSA strain (Fig. 8). Collectively, these results validate our screen data that had suggested pronounced competitive capacity of *S. hominis* S34-1 and indicate potential in a probiotic-type of topical application to reduce skin disease by *S. aureus*.

### Development of MP1 nanoparticle formula

**Fig. 9:**
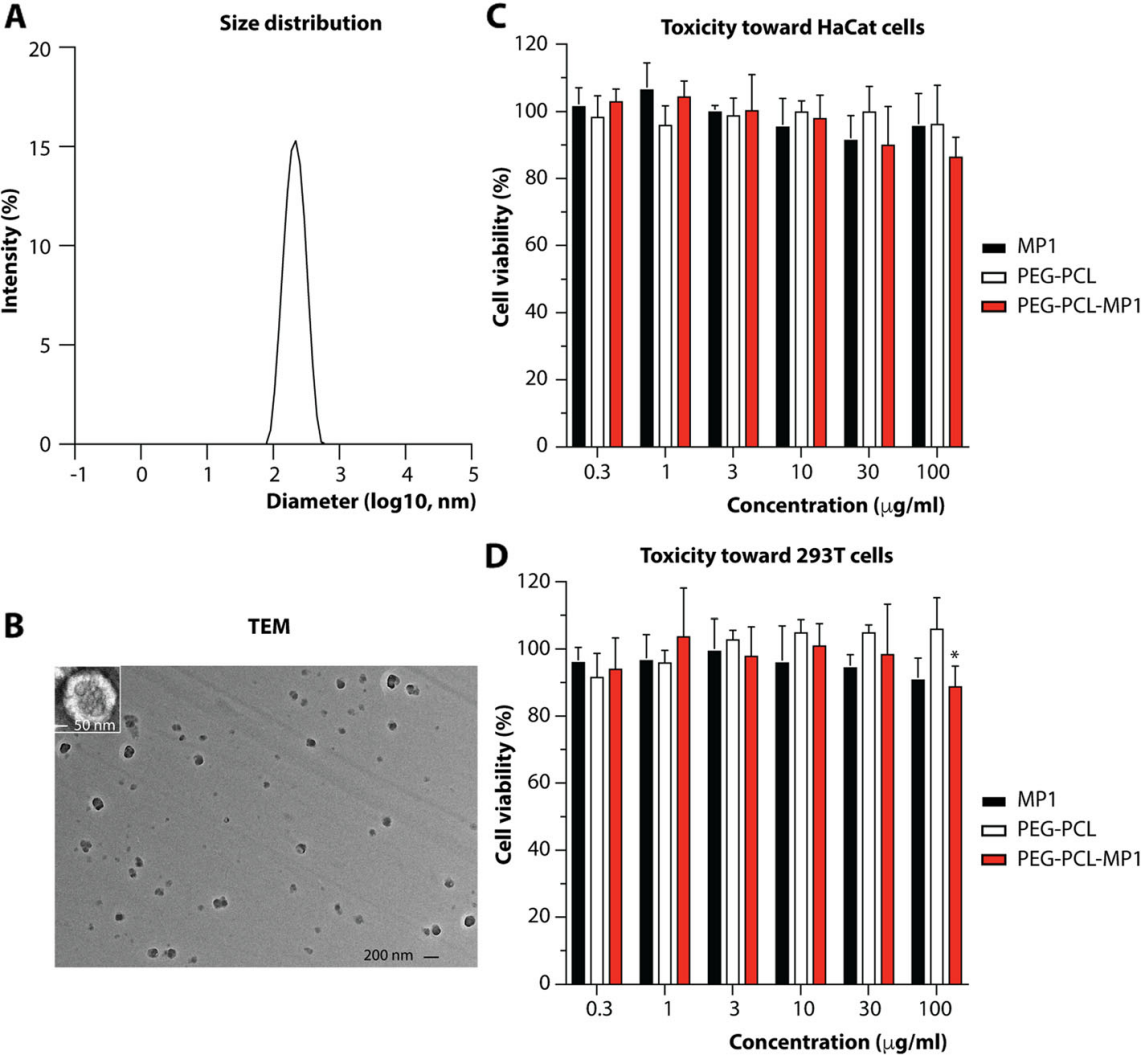
Characterization of PEG-PCL-MP1 micelles. Size distribution **(a)** and transmission electron microscopy (TEM) image **(b)** of MP1-loaded PEG-PCL micelles. **(c, d)** Toxicity tests toward HaCaT **(c**) and 293 T cells **(d).** Cell viability was determined using a cell counting kit (CCK)-8 assay with treatment by free MP1 and PEG-PCL-MP1 at the same MP1 dose and MP1-free PEG-PCL micelles with the same micelle amount as PEG-PCL-MP1 and with incubation for 24 h. Statistical analysis is by 2-way ANOVA. The only statistically significant difference found is marked by an asterisk (*p* = 0.028). Error bars show the mean ± SD

MP1 has been described to exert remarkable antitubercular [30], antiviral [31], and antibacterial activities [32], but poor water solubility due to considerable hydrophobicity and associated low bioavailability have prevented further development into an effective anti-infective [33]. We hypothesized that by binding MP1 to polymeric micelle-type nanoparticles, we could achieve a nanoparticle MP1 formula to overcome the problems associated with the physico-chemical properties of MP1. Such micelle-based nanoparticles, self-assembled colloidal particles with a hydrophobic core and hydrophilic shell, are used for the delivery of poorly soluble drugs for example in cancer therapy [34]. We here used a combination of the well-established polyethylene glycol (PEG) [35] and polycaprolactone PCL [36] components to form the hydrophilic shell and hydrophobic core, respectively, of the nanoparticle micelles. PEG-PCL micelles are known to exhibit the considerable advantages of being amphiphilic and able to release drugs at a controlled rate [37, 38]. The MP1-loaded PEG-PCL micelles we produced had homogeneous spherical shapes, an average size of 188.2 nm, and a polymer dispersity index of 0.16 (Fig. 9a and b). They lacked toxicity as tested against keratinocyte (HaCaT) and embryonic kidney (293 T) cells up to a concentration of 30 μg/ml (Fig. 9c and d).

### In vitro antimicrobial activity of PEG-PCL-MP1 nanoparticles

**Fig. 10:**
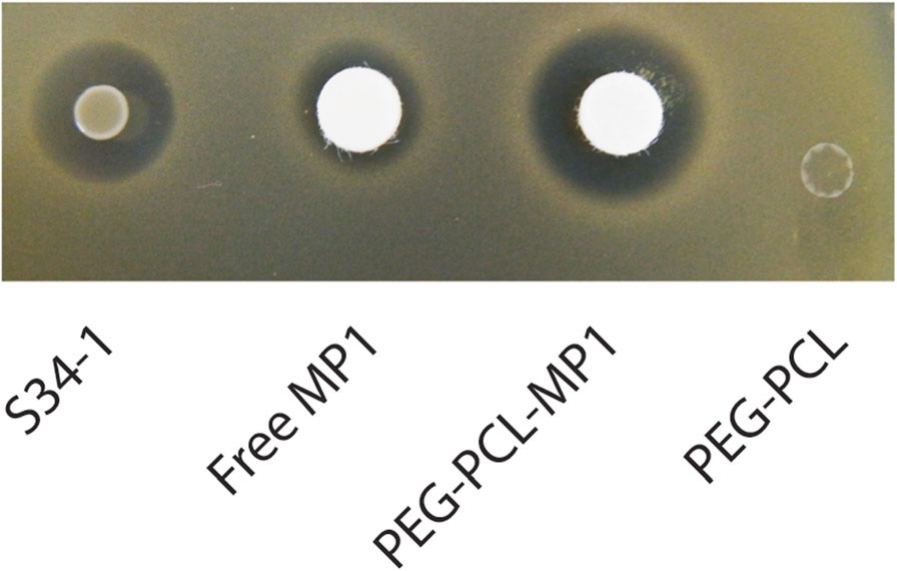
In vitro antimicrobial activity of PEG-PCL-MP1 nanoparticles. A total of 10 μl of PEG-PCL-MP1 nanoparticles (200 ng/μl) were spotted onto *S. aureus* test plates and compared to MP1 at the same concentration. *S. hominis* S34-1 bacteria and bare PEG-PCL (40 μg) were also spotted as controls

To test the antimicrobial activity of the PEG-PCL-MP1 nanoparticle formula compared to free MP1, we used an agar diffusion assay on agar plates with embedded *S. aureus* (Fig. 10) and performed broth dilution tests to determine the MIC (Table 3). The PEG-PCL-MP1 nanoparticles showed considerably stronger activity than free MP1 in both assays.

**Table 3:** MIC determination of MP1 and PEG-PCL-MP1 nanoparticles against *S. aureus*

Strains	MIC of PEG-PCL-MP1 (μg/ml)	MIC of free MP1 (μg/ml)
ST59	0.125	0.5
ST8	0.125	0.5
ST5	0.25	1
ST239	0.125	0.5
ST398	1	> 1^a^

^a^Solubility of MP1 is ~1 μg/ml, so higher concentrations could not be tested

### Treatment of local and systemic *S*. *aureus* infection by PEG-PCL-MP1 nanoparticles

To test whether MP1-loaded PEG-PCL nanoparticles are efficient in reducing local and systemic infection with *S. aureus*, we used a skin abscess model of local infection, as well as two systemic infection models, one with a high-infecting dose to test for reduction of mortality, and a lower-dose model to analyze for reduction of other disease manifestations. All models were performed with a virulent MRSA strain and several control groups, which in addition to the MRSA-only and PEG-PCL-MP1-formula-treated groups, included a PBS-only, a MRSA-infected/MP1 only-treated, and a MRSA-infected/PEG-PCL only-treated control.

**Fig. 11:**
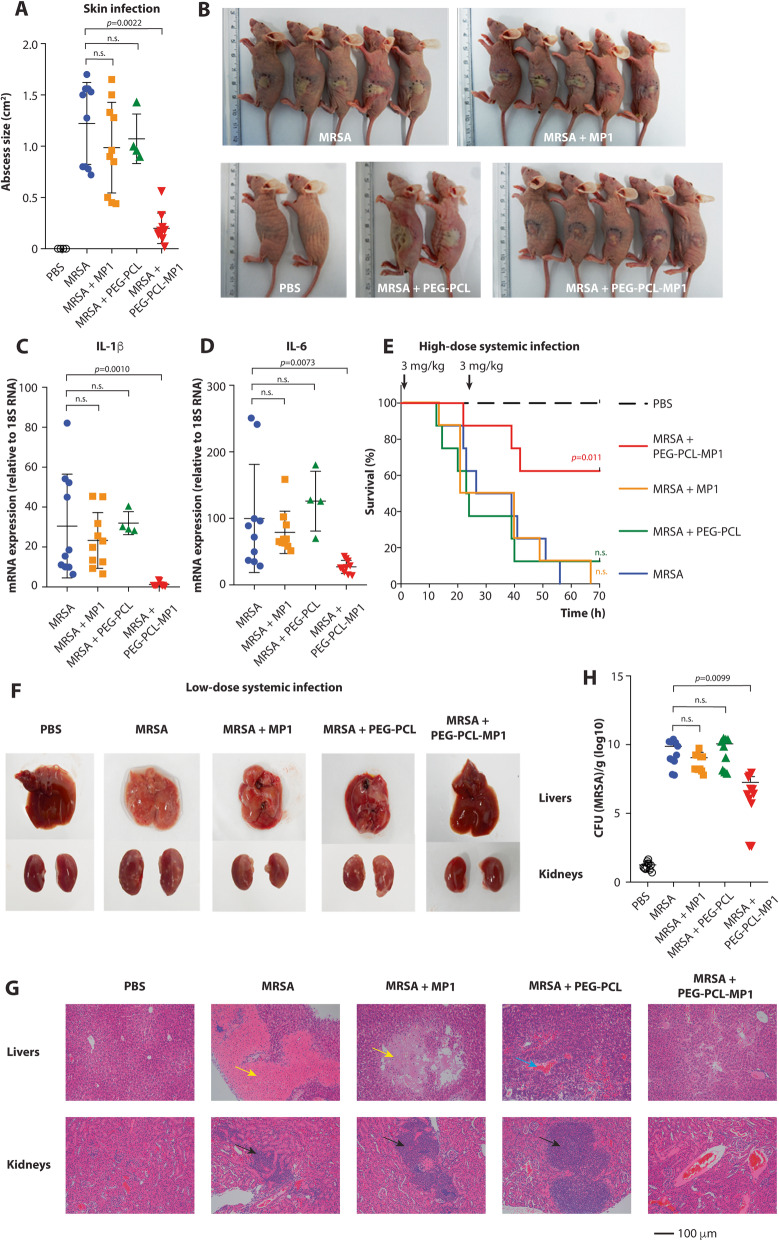
Application of PEG-PCL-MP1 nanoparticle treatment for MRSA infection. **a–d** Skin infection. *n* = 5 mice/group, except PBS and MRSA + PEG-PCL control groups, *n* = 2. Two abscesses per mouse were produced. **a** Abscess sizes measured 22 h post-infection with 1 × 10^8^ CFU MRSA (ST59) and the indicated treatment. **b** Abscess pictures. **c, d** Levels of cytokine gene expression in 22 h-abscesses determined by qRT-PCR. **e** High-dose systemic infection (sepsis), survival curves. Animals (*n* = 8/group) received 5 × 10^7^ CFU MRSA (ST59) and the indicated treatment (3 mg/kg at 1 and 24 h). Analysis is by log-rank (Mantel-Cox) tests of the respective curves versus the MRSA curve. **f–h** Low-dose systemic infection (bacteremia). Animals (*n* = 6/group) received 1 × 10^7^ CFU MRSA (ST59) and the indicated treatment. Animals were sacrificed 48 h post-infection. **f** Macroscopic analyses of livers and kidneys. Representative organs are shown. **g** Histopathological analysis of liver and kidney tissues. Representative pictures are shown. Arrows indicate tissue necrosis (yellow), hemorrhage (blue) and massive infiltration of inflammatory cells (black). **h** Bacterial load in the kidneys. **a, c, d, h** Statistical analysis is by Kruskal-Wallis tests and Dunn’s post-tests. Error bars show the mean ± SD.

In the skin abscess model, the application of PEG-PCL-MP1 nanoparticles strongly reduced the pronounced abscess formation and concomitant inflammation (as measured by levels of the cytokines IL-1β and IL-6) observed in the MRSA-infected group, while the application of MP1-only or PEG-PCL nanoparticles only had no effect (Fig. 11a–d). In the high-dose systemic infection model, there was a significant decrease in mortality when mice received an injection of PEG-PCL-MP1 nanoparticles, which was not the case with any of the control treatments (Fig. 11e). In the lower-dose systemic infection model, the formation of liver and kidney abscesses was reduced upon macroscopic observation in the PEG-PCL-MP1 nanoparticle-treated but not the control groups (Fig. 11f). By microscopy, kidney tissue showed less inflammation and liver tissue did not exhibit features of necrosis or hemorrhage, as compared to the control groups (Fig. 11g). Furthermore, the bacterial load in the kidneys was measured and found to be significantly decreased in the PEG-PCL-MP1 nanoparticle-treated but not the control groups (Fig. 11h). These results show that PEG-PCL-MP1 nanoparticle treatment significantly reduces *S. aureus* local and systemic infection. Notably, they demonstrate that micelle nanoparticle formulation of MP1 overcomes the previously noted therapeutic limitations of unmodified, hydrophobic MP1, which were reflected in our results.

## Discussion

In the present study, we used a drug discovery approach that is based on information derived from competitive phenomena between microorganisms. However, in contrast to most previous approaches that have commonly analyzed environmental samples, we used a systematic analysis of the human microbiota for that purpose. We reasoned that in comparison to environmental sampling, an analysis of the human microbiota is more likely to discover strains and substances that are effective against human pathogens. We screened the microbiota of the human skin as an example, prompted by the fact that one of the most dangerous and multi-drug resistant bacteria, *S. aureus*, lives on human skin.

Focusing on bacterial commensals, our approach was prone to identify bacteriocins. The main differences between bacteriocins and antibiotics are their peptide-based nature and a frequently narrower target spectrum. Nevertheless, bacteriocins from Gram-positive bacteria, for example, often are active toward a wide variety of other Gram-positive bacteria. With the number of new antibiotics in development still being dangerously low, bacteriocins have recently moved closer to the center of interest in anti-infective drug development [39]. We isolated as the most promising candidate from our screen a strain of *S. hominis* with broad competitive activity against Gram-positive bacteria, including against major pathogens, two of which, MRSA and VRE, are listed among the most serious antibiotic-resistant “ESKAPE” pathogens. The active substance was identified as MP1, a thiopeptide previously described to have antitubercular, antimalarial, and antibacterial effects and to act by binding to the L11 binding domain of the 23S ribosomal RNA, thereby inhibiting translation [40].

Our study describes two exemplary strategies of how the study of the human microbiota and subsequent isolate selection from that source can be used for anti-infective drug development. First, the most direct translation of our finding into a potential treatment option would consist in using the considerable competitive capacity that *S. hominis* S34-1 exhibited against *S. aureus* to treat *S. aureus*-caused abscesses and *S. aureus*-infected chronic wounds, which occur particularly in diabetic patients, in a “probiotic” approach using topical application of live bacteria [41]. Given the broad target spectrum of *S. hominis* S34-1 and MP1 against a series of other Gram-positive pathogens, many of which also contribute to mixed wound infections [41] or cause severe skin disease [42], these findings indicate the potential use of *S. hominis* S34-1 beyond against only *S. aureus*. Furthermore, our data suggest *S. hominis* S34-1 may also be used for *S. aureus* decolonization purposes. Despite having so far been tested only with antibiotics as decolonization agents, this general approach is often suggested for the prevention of *S. aureus* infections in certain parts of a population [43]. Using a beneficial, competitive commensal for that purpose would overcome the problems that are associated with the considerable use of antibiotics, such as most notably the development of antibiotic resistance.

Second, we hypothesized that with the more traditional way of using the identified antibacterial substance in pure form, allowing the systemic application, one might be able to combat a variety of infection types. However, as is the case with many natural substances, considerable hydrophobicity of MP1 has hitherto prevented its further development into a potential anti-infective drug [44]. We overcame this problem by using an MP1-loaded nanoparticle micelle formula, whose application in contrast to pure MP1 significantly decreased MRSA-induced signs of disease and mortality in systemic and skin infection. We believe this method to be broadly applicable for the development of many of the frequently hydrophobic bacteriocins into potential drugs.

Our study also revealed an interesting insight into age-dependent changes in the bacterial composition of the skin microbiota. Staphylococci dominated most in young adults and the abundance of *S. aureus* as compared to CoNS was strongly diminished in that age group as opposed to in children and seniors. However, the low absolute numbers of *S. aureus* we found in general precluded meaningful statistical analysis of that difference. Young adults are strongly immunocompetent, which may affect bacterial composition in yet unknown ways and lead to a lower abundance of pathogens. While unrelated to the purpose of the present study, the mechanism underlying those phenomena certainly warrant further investigation.

## Conclusions

Our findings indicate a potential for probiotic and systemic application of *S. hominis* S34-1 and PEG-PCL-MP1 nanoparticles, respectively, in the treatment of *S. aureus* infections or *S. aureus* decolonization and provide an example of how analysis of commensal bacteria can be exploited for drug discovery. While the determined target range of MP1 contains a series of Gram-positive pathogens, *Mycobacterium tuberculosis*, and viruses, suggesting much broader applicability, we have not yet tested its value in treating infections with other pathogens. Further limitations of our study include that so far, we have not tested for potential deleterious effects of *S. hominis* S34-1 in a live, probiotic form in situations with underlying complicating conditions, such as a compromised immune system or presence of indwelling medical devices that may become infected with CoNS. Finally, while initial toxicity tests are promising, more detailed phase 1 tests, such as pharmacokinetic tests, will have to be performed before moving our formula to clinical tests.

## Methods

### Study design

This study consisted of a sample collection part, in which bacterial isolates were obtained from healthy human volunteers, typed and screened using laboratory tests for antimicrobial activities. The human subjects were pre-selected to fit in specific age groups and were subject to the exclusion criteria listed below. The other part of the study comprised animal models and controlled laboratory experiments aimed to identify the produced antimicrobial substance and determine its capacity, or that of the producer strain, to reduce *S. aureus* infection or colonization. Details are listed in the respective sections. No blinding was used during group allocation. No outliers were excluded.

### Participant enrollment and skin swab collection

Three groups of healthy volunteers were recruited from the community in Shanghai, China. In total, 156 children (5–6 years old), 210 young adults (18–20 years old), and 160 seniors (50–90 years old) were enrolled for the research. Routine physical examination and health status enquiries were performed. The exclusion criteria for the study were as follows: abnormal physical examination results, pregnancy or breast-feeding, diabetes or other chronic metabolic diseases, various cancers, history of dermatitis or prior chronic skin disorders, carriage of medical devices, contact with the hospital environment in the last six months preceding enrollment, use of any antimicrobials within 1 month preceding enrollment. Skin swabs were collected from the cubital fossae of each participant. Swabs were immediately submerged in 1 ml sterile saline for further use.

### Bacterial strains, growth conditions, and plasmids

The indicator strains for the screen of antimicrobial activities from human isolates are listed in Table 1. The Gram-positive pathogens used to test the activity of *S. hominis* S34-1 are listed in Table 2. The MRSA strain used in all experiments, except for the experiment that used luminescence to detect bacteria in vivo, was the community-associated (CA)-MRSA strain ST59 (RJ-2). The bioluminescent strain was *S. aureus* Xen36, which is derived from *S. aureus* ATCC 49525 and possesses a stable copy of the modified *Photorhabdus luminescens luxABCDE* operon at a single integration site on a native plasmid [45]. The transposon mutant Tn1, which has a transposon insertion in the structural MP1 gene *tclE* was used as the S34-1 mutant strain (S34-1Δ). For transposon mutagenesis, plasmid pBTn was used, which contains a 1.45-kb *ermB* fragment of Tn551 between the *himar1* inverted repeats and the *himar1* mariner transposon under the control of a xylose-inducible promoter [23]. Bacteria were generally grown in tryptic soy broth (TSB; Oxoid) with shaking at 200 rpm at 37 °C.

*S. hominis* growth curves were recorded using a microtiter plate reader (Synergy 2, Biotek) by measuring the absorbance at 600 nm. Overnight cultures of *S. hominis* S34-1 and S34-1Δ were diluted in 10 ml fresh TSB to an OD_600_ of 0.03 and shaken at 200 rpm at 37 °C. OD_600_ was measured every hour up to 10 h.

### Bacterial identification in skin swabs

To identify bacteria in human skin samples, skin swabs were submerged in 1 ml sterile saline and vortexed for 2 min. A 100-μl sample from each swab was plated on 5% sheep blood agar and incubated at 37 °C for 24 h. In almost all swab samples, 12 colonies or fewer grew, and all colonies were subjected to species identification. In the skin swab samples that grew more than 12 colonies, 12 colonies were randomly selected. Species identification for staphylococci was performed using matrix-assisted laser desorption ionization time-of-flight mass spectrometry (MALDI-TOF-MS; Bruker Daltonics). Briefly, a single clone was spotted onto the steel target plate, 1 μl 10% formic acid (Sigma) was added and dried for 5 min at 75 °C. The spot was overlaid with 1 μl MALDI matrix (a saturated solution of α-cyano-4-hydroxycinnamic acid in 50% acetonitrile-2.5% trifluoroacetic acid, Fluka). After drying, the plate was subjected to MALDI-TOF MS analysis. Spectra were obtained in linear positive-ion mode range from 2000 to 20,000 Da. Each spot was measured on five different positions by using 1000 laser shots at 25 Hz in groups of 40 shots. The MALDI Bruker Biotyper 3.0 software and library (Bruker Daltonics) were used for spectra analysis according to the spectra matching and score criterion. PCR amplification and 16S rRNA sequencing using primers F: 5’-AGTTTGATCCTGGCTCAG-3’ and R: 5’-GGTTACCTTGTTACGACTT-3’ were used to identify isolates that failed to be confirmed by MALDI-TOF-MS.

Multilocus sequence typing of the *S. hominis* isolates was determined by amplifying the six housekeeping genes *arcC*, *glpK*, *gtr*, *pta*, *tpiA*, and *tuf* as described previously. The determination of alleles was performed using the MLST website (http://www.mlst.net) for *S. hominis.*

### Screening for antimicrobial activity by agar diffusion assay

All tested *S. hominis* and *S. epidermidis* isolates were cultivated in TSB at 37 °C with shaking at 200 rpm for 12 h. To prepare test plates of *Staphylococcus*, *Enterococcus*, and *Streptococcus* species (except *S. pneumoniae* and *S. constellatus*), Gram-negative bacteria, and *Micrococcus luteus*, overnight cultures were diluted in melted TSA (Oxoid) to an OD_600_ of 0.001 (except for *Enterococcus* strains for which OD_600_ = 0.01 was used) and then poured into Petri dishes. For other bacterial species, which grew poorly on TSA plates, the test was performed after first applying a lawn of the indicator strain at an OD_600_ of 0.1 to the surface of a blood agar plate. To obtain concentrated supernatant of *S. hominis* S34-1, an overnight culture was diluted in a tube containing 10 ml fresh TSB to an OD_600_ of 0.03. After shaking for 24 h at 200 rpm and 37 °C, the tube was centrifuged at 5,000×*g* for 10 min. The supernatant was sterile-filtered twice using 0.22-μm filters (Millipore) and put in a freeze dryer at −110 °C for 48 h. This resulted in yellowish powder, which was then dissolved in PBS to 1 μg/μl and stored at 4 °C. A total of 2-μl spots of this solution were applied to each bacterial lawn. The plates were incubated at 37 °C for 16–24 h (except for *C. acnes*, which was cultured anaerobically at 37 °C for 48 h) and evaluated for inhibition zones.

### Whole-genome sequencing

Whole-genome sequencing was performed on a PacBio platform after a quality control process that involved disposing of raw data by removing adapter sequence and non-A, T, C, G bases at the 5’ end, trimming low-quality sequencing reads, discarding reads with ‘N’ base percent beyond 10% and fragments less than 25 bp. Coding genes were identified by Glimmer3. tRNA and rRNA were predicted by tRNAscan-SE software and Barrnap software, respectively. The annotation process was finished by two steps, one of which was constructing a library by formatdb software and another was BLAST comparison.

### Transposon mutagenesis of *S. hominis* S34-1

Transposon mutagenesis was performed using plasmid pBTn, which contains a xylose-inducible Himar1-transposase gene. The pBTn plasmid was first transferred into *S. hominis* S34-1 by electroporation as described [46] using high amounts (μg range) of plasmid DNA. Then, the resulting *S. hominis* S34-1 (pBTn) clone was inoculated 1:100 from an overnight culture in 10 flasks each containing 500 ml fresh TSB [containing 0.5% xylose (Sigma), 10 μg/ml chloramphenicol (Cm10, Sangon), and 5 μg/ml erythromycin (Em5, Sigma-Aldrich)]. Flasks were then shaken in a shaking incubator at 200 rpm and 30 °C. On the next day, 5 ml from each bottle was transferred into 500 ml fresh TSB (Em5, no glucose) of another 10 bottles, which were grown at 200 rpm and 43 °C, and this process was repeated at least twice. Aliquots from each bottle were plated onto TSA/Em5. The putative transposon insertion mutants (Em^r^ and Cm^s^) were selected by streaking the Em^r^ colonies onto TSA/Cm10 and were stored at −80 °C for library screening. The four clones that showed the desired phenotype were also confirmed by multi-locus sequence typing (MLST) to be derived from the S34-1 wild-type strain.

### Library screening, inverse PCR, and nucleotide sequencing

Single clones were inoculated into fresh TSB in 96-well deep-well plates, grown at 37 °C overnight with shaking at 200 rpm, and then aliquots were dotted on an MRSA lawn. DNA of desired mutants (those not showing inhibition zones) was extracted and subjected to inverse PCR as described [23]. Nucleotide sequence analysis was performed by sequencing the product from the second PCR. Insertion sites were determined by aligning the flanking region with the *S. hominis* S34-1 whole-genome sequence.

### Production, analysis, and purification of the antimicrobial compound

For purification of the antimicrobial activity, *S. hominis* S34-1 was grown in liquid TSB medium at 37 °C with shaking at 200 rpm for 24 h. For large-scale fermentation, aliquots (~10 ml) of the seed cultures were transferred into seven 2-l Erlenmeyer flasks each containing 700 ml of TSB medium (4.9 l in total) and incubated at 37 °C with agitation at 200 rpm for 24 h. These broths were then combined and centrifuged at 5000×*g* for 10 min. The supernatant was extracted with ethyl acetate three times. The organic layers were then combined and concentrated in vacuo to yield a yellow gum.

Further purification was performed using repeated reversed-phase column chromatography. In vacuo concentrated extracts were dissolved in methanol and injected onto an Agilent ZORBAX Eclipse XDB-C18 reversed-phase column (5 μm pore size, 9.4 × 250 mm), and eluted with a gradient as follows (H_2_O as eluent A, and acetonitrile as eluent B): 0–20 min, 35–65% B; 20–28 min, 65–95% B; 28–30 min, 95% B at a flow rate of 1.0 ml/min. The peak eluting at a retention time of 14.6 min was collected. The yield from 4.9 l culture was ~3.1 mg of the compound in pure form. NMR and HPLC/ESI-MS spectra were recorded for the structural elucidation of the compound.

### 1D-NMR and HPLC/ESI-MS analyses

^1^H-NMR spectra were collected on an Agilent 400-MR DD2 NMR spectrometer. Chemical shifts were reported in ppm using tetramethylsilane as an internal standard. HPLC/ESI-MS analysis of the purified compound was carried out on a Thermo Electron LTQ-Orbitrap XL mass spectrometer connected to an Agilent HP 1100 HPLC system. All solvents used were of analytical grade purchased from J. T. Baker.

### Encapsulation of MP1 with PEG-PCL

PEG (2 K)-PCL (5 K)-aldehyde and PCL (2 K)-PEG (2 K) (Resenbio) were dissolved in chloroform to a final concentration of 5 mg/ml at a mass ratio of 7:3. MP1 dissolved in chloroform was mixed with that PEG-PCL solution to reach a drug-loading capacity of 5%. The mixture was then transferred to a round-bottom flask to which the same volume of highly purified water was added, followed by complete ultrasonic emulsification. The chloroform was then evaporated in vacuo and the residue was dialyzed against PBS for 12 h to disperse the formed micelles. During this process, the amphiphilic PEG-PCL copolymers self-assemble into a molecular preparation possessing a hydrophobic inner core and a hydrophilic shell. Due to its hydrophobicity, MP1 partitions into the hydrophobic core of the micelles.

### Characterization of PEG-PCL-MP1 micelles

The size of the micelles was determined in cuvettes using a Zetasizer Nano ZS90 (Malvern) instrument. Micelle morphologies were analyzed by transmission electron microscopy (TEM) using a Hitachi HT770 electron microscope operating at 100 kV. To that end, copper TEM grids were covered with a layer of carbon to deposit the sample. The micelle solution was then dropped on the grid and dried for about 20 min. The sample was analyzed on the microscope unstained or negatively stained. For negative staining, a drop of phosphotungstic acid (2 %) was added to the sample to stain for 1–3 min. Excess deposits were absorbed by filter paper and the sample was air-dried.

### Cytotoxicity assay

We used two cell lines to analyze the toxicity of PEG-PCL-MP1 micelle nanoparticles*.* The 293 T cell line is a derivative of human embryonic kidney 293 cells. HaCaT is an immortal keratinocyte cell line from adult human skin. For the toxicity assays, cells were grown at 37 °C in a 5% CO_2_ incubator in a medium consisting of Dulbecco’s modified Eagle’s medium (Gibco) supplemented with 10% fetal bovine serum (Gibco) and 1% penicillin/streptomycin (Sigma). Cell pellets were suspended in fresh medium and cell density was adjusted to 2 × 10^5^ cells/ml for 293 T cells and 1 × 10^5^ cells/ml for HaCaT cells. Cells were seeded in 96-well plates at 0.1 ml/well and pre-cultured for 24 h. Then, cells were exposed to different concentrations of bare PEG-PCL (PEG-PCL concentration of 6, 20, 60, 200, 600, and 2000 μg/ml), MP1-loaded PEG-PCL and pure MP1 (MP1 concentrations of 0.3, 1, 3, 10, 30, and 100 μg/ml) for 24 h. Triplicate wells for each group were set up and wells with untreated cells served as a negative control. After removing the culture medium, 100 μl fresh medium was added. The Cell Counting Kit (CCK)-8 (Yeasen) assay was performed by adding 10 μl of WST-8 [2-(2-methoxy-4-nitrophenyl)-3-(4-nitrophenyl)-5-(2,4-disulfophenyl)-2H-tetrazolium, monosodium salt] solution to cells and incubating for 1 h at 37 °C under 5% CO2. Finally, cell viability was measured on a Synergy 2 microplate reader (Biotek) at 450 nm and expressed by the following equation: cell viability (%) = (Abs_sample_−Abs_blank_) /(Abs_control_−Abs_blank_) × 100. The assay was repeated three times.

### RNA extraction and quantitative real-time polymerase reaction (qRT-PCR)

Total RNA was extracted using RNeasy® kits (Qiagen) according to the manufacturer’s instructions, checked for purity, and concentration was assessed using a NanoDrop spectrophotometer (Thermo Scientific). A total of 0.5 μg RNA was reverse-transcribed to cDNA using a QuantiTect® kit (Qiagen). For real-time PCR, which was performed on a 7500 real-time PCR system (Applied Biosystems), each 25 μl reaction contained 12.5 μl SYBR green mix (Roche), 0.1 μl of 100 μM of each primer (Table 4), 9.8 μl highly purified H_2_O, and 2.5 μl template. mRNA expression relative to 18S rRNA was calculated using the 2^−ΔΔCT^ method.

**Table 4:** Primers for qRT-PCR

Target genes	Orientation	Sequence (5’-3’)
IL-6	Forward Reverse	CTGCAAGAGACTTCCATCCAGTT GGGAAGGCCGTGGTTGTC
IL-1β	Forward Reverse	TGCAGAGTTCCCCAACTGGTACATC GTGCTGCCTAATGTCCCCTTGAATC
18S	Forward Reverse	CATTCGAACGTCTGCCCTATC CCTGCTGCCTTCCTTGGA

### RNA-SEQ

Bacterial cells of *S. hominis* S34-1 wild-type and S34-1Δ mutant with the same initial OD600 of 0.03 were grown at 37 °C with shaking at 200 rpm for 4 h and harvested by centrifugation. After washing cells with sterile PBS, RNA extraction was performed as described above. For the removal of rRNA from samples, the Ribo-Zero rRNA Removal Kit (Illumina) was used. After synthesizing cDNA, 3′ ends of the DNA fragments were adenylated and ligated with Illumina PE adapter oligonucleotides. The libraries were purified using the AMPure XP system (Beckman Coulter) and then selectively enriched using Illumina PCR Primer Cocktail. Products were purified with an AMPure XP system and quantified using the Agilent high sensitivity DNA assay on a Bioanalyzer 2100 system (Agilent). The RNA-seq libraries were then sequenced on a NextSeq 500 platform (Illumina). The transcriptome analysis was performed using CLC Genomics Workbench 8.0 (CLC BioQiagen). The expression values were then analyzed using a DESeq package of R/Bioconductor. Differentially expressed genes were determined by performing a negative binomial test using the DESeq software, with thresholds of *p* value < 0.05 and absolute fold change ≥ 2.

### Animal studies

Outbred, immunocompetent hairless mice were used for the abscess model. BALB/c female mice were used for the colonization and wound healing model. ICR mice were used in the bacteremia model with PEG-PCL-MP1 treatment. All mice were between 4 and 6 weeks of age at the time of use and had access to food and water ad libitum.

### Mouse skin punch competition model

MRSA ST59 and *S. hominis* S34-1 wild-type and mutant S34-1Δ were grown overnight and then subcultured (1:100) in fresh TSB and grown for 4 h with shaking at 200 rpm and 37 °C. Cells were harvested by centrifugation at 5000×*g* for 10 min and washed twice with sterile PBS. To determine bacterial growth, mice were divided into two groups of 18 mice each. Hair was removed the day before surgery. On the day of surgery, mice were anesthetized. A sterile 4-mm biopsy punch was used to outline a circular pattern on the left flank of each mouse, which was cut using scissors and inoculated with bacteria (S34-1 or S34-1Δ 1 × 10^9^ CFU/ml in 10 μl of PBS). At time points 4 h, 8 h, and 24 h, six mice in each group were anesthetized and 8-mm punch biopsy specimens of lesional skin were taken and subsequently homogenized in PBS using a manual homogenizer (Tiangen). Appropriate dilutions of the samples were plated on blood agar and the CFU of *S. hominis* were counted after incubating the plates at 37 °C for 24 h.

For treatment experiments, mice were placed in individually ventilated cages as three treatment groups comprising 17 mice per group. The day before, the posterior lower backs of mice were clipped free of fur. Sterile square silicone sheets of one square centimeter were prepared and a 5-mm-length square hole was cut in the center of each sheet. On the day of operation, mice were anesthetized by 2,2,2-tribromoethanol (Sigma-Aldrich). One flank of the back of each mouse was swabbed with 70% ethanol and then pasted with a square sheet. A total 5 μl of one the following inocula was pipetted slowly into the holes: (i) 1 × 10^7^ CFU MRSA; (ii) 1 × 10^7^ CFU MRSA and 1 × 10^7^ CFU S34-1; (iii) 1 × 10^7^ CFU MRSA and 1 × 10^7^ CFU S34-1Δ. After the hole area dried, the fur-free backs were covered with OpSite dressings. Twenty-four hours later, skin samples were taken with an 8-mm-diameter punch, the tissue samples were homogenized in PBS using a manual homogenizer (Tiangen), and CFU of MRSA (which is hemolytic in contrast to *S. hominis*) were determined by plating ground skin tissue samples suspended in 100 μl PBS on blood agar and evaluating the hemolytic capacity of colonies after incubating the plates at 37 °C for 24 h.

### Mouse skin abscess model

Overnight cultures (grown in TSB) of the strain CA-MRSA ST59 were subcultured (1:100) in fresh TSB for and grown for 4 h. After centrifugation at 5000×*g* for 10 min, cells were washed twice and resuspended in PBS. Mice were anesthetized with 2,2,2-tribromoethanol (Sigma-Aldrich) and subcutaneously inoculated with 0.1 ml live cells on the left and right flanks. For bacterial CFU determination, the transiently swelled area was marked by outlining. At time points 4 h, 8 h. and 24 h, the same number of mice were anesthetized and the marked skin area was cut with an 8-mm-diameter punch. The tissue samples were homogenized in PBS using a manual homogenizer (Tiangen), and CFU of *S. hominis* was determined by plating ground skin tissue samples in appropriate dilution series on blood agar after incubating the plates at 37 °C for 24 h.

Abscess development was monitored by measuring abscess sizes daily for a period of 8 days. The abscess length (L) and width (W) dimensions were used to calculate the abscess area using the formula L × W. Tissue samples from each group were fixed in 4% formalin (Sangon) for 24 h at 4 °C, embedded in paraffin, sectioned, stained with hematoxylin and eosin (HE), and photographed at × 100 magnification by light microscopy.

For the PEG-PCL-MP1 treatment model, each skin flank received treatments with 10 μg of PEG-PCL-MP1, bare PEG-PCL micelles, or free MP1 via intra-abscess injection 1 h after MRSA inoculation. Skin abscesses were measured as described above. Abscesses were excised 22 h post-infection and homogenized to extract RNA for measurement of inflammatory cytokine gene expression.

### Mouse wound healing model

The model was performed essentially as previously described [47, 48]. Mice were divided into four treatment groups with two mice per cage, and each group contained four mice. Hair was removed the day before surgery. On the day of surgery, mice were anesthetized and one full-thickness excisional wound on the left flank of each mouse was created. The wounds were subsequently inoculated with bacterial suspension as follows: (i) 1 × 10^7^ CFU of Xen36; (ii) Xen36 and S34-1 at 1 × 10^7^ CFU each; (iii) Xen36 and S34-1Δ at 1 × 10^7^ CFU each; (iv) 1 × 10^7^ CFU of S34-1. Skin wounds were photographed with an in vivo imaging system on days 1, 3, 8, and 15 to measure the bacterial burden.

### Systemic infection models

An overnight culture of CA-MRSA ST59 was sub-cultured (1:100) in fresh TSB and grown for 4 h. Then, cells were harvested by centrifugation at 5000×*g* for 10 min, washed twice with PBS, and resuspended in PBS at 1 × 10^8^ CFU/ml. A total of 100 μl (1 × 10^7^ CFU of MRSA) of that suspension was then injected intravenously into the animal via the orbital vein. After 1 h, a dose of 2 mg/kg of PEG-PCL-MP1, free MP1, or bare PEG-PCL was given via tail-vein injection. Equivalent doses were given every 12 h for the duration of the experiment. The untreated group received 100 μl of PBS at the same time points instead. Mice were weighed before bacterial challenge and for two consecutive days after challenge. At 48 h post-infection, the animals were euthanized, and their kidneys were collected in tubes containing 1 ml PBS. The tissues were homogenized on ice using a manual homogenizer (Tiangen) and appropriate dilutions were prepared and plated for the determination of bacterial loads. Tissue sections were fixed in 4% formalin and processed for routine histopathology.

The survival (high-dose) model was performed analogously to the above-described model, but with an inoculum of 5 × 10^7^ CFU of MRSA and only one repeated-dose 24 h after the first dose, both of which at 3 mg/kg. The survival status of mice was recorded at different intervals up to 70 h.

### Statistical analysis

Data for the classification of skin bacterial strains and inhibition zones (heatmap) were analyzed using R (version 3.4.4). For in vitro and animal studies, statistical analyses were performed using GraphPad Prism 8 software. Data were analyzed for equal distribution using Anderson-Darling, D’Agostino & Pearson, Shapiro-Wilk, and Kolmogorov-Smirnov tests. If all groups were equally distributed by results from all four tests, 1- or 2-way ANOVAs were used, as appropriate, to analyze the data. Otherwise, non-parametric tests (Kruskal-Wallis) were used. Survival curves were analyzed by log-rank (Mantel-Cox) tests. Correlative analyses were performed using chi-square tests. Values of *p* < 0.05 were considered to be statistically significant.

## Supplementary information

**Additional file 1: Table S1.** Bacteria isolated in this study

**Additional file 2: Table S2**. RNA-Seq results of *S. hominis* S34-1 wild-type versus S34-1Δ comparison
