# Supplementary material for: Skin microbiota analysis-inspired development of novel anti-infectives
Source: Microbiome. 2020 Jun 5;8:85. doi: 10.1186/s40168-020-00866-1 (PMC7275423; doi:10.1186/s40168-020-00866-1)
Supplement: Supplementary file 2 — Additional file 1: Table S1. Bacteria isolated in this study [file 40168_2020_866_MOESM1_ESM.pdf]

Additional Table 1. Bacteria isolated in this study.

	Children	Adults	Seniors
Acinetobacter baumannii	3	0	1
Acinetobacter johnsonii	0	0	4
Acinetobacter junii	0	0	1
Acinetobacter lwoffii	2	1	4
Acinetobacter parvus	0	0	1
Acinetobacter pittii	3	0	0
Actinomyces neuii	0	0	1
Actinomyces oris	20	0	3
Aerococcus viridans	0	1	2
Arthrobacter cumminsii	0	0	4
Arthrobacter histidinolovorans	0	0	1
Arthrobacter sp.	0	0	2
Bacillus arsenicus	1	1	2
Bacillus cereus	4	3	0
Bacillus circulans	1	0	0
Bacillus endophyticus	1	0	0
Bacillus firmus	1	0	0
Bacillus horneckiae	1	0	0
Bacillus idiensis	0	1	0
Bacillus infantis	1	0	0
Bacillus kochii	2	0	0
Bacillus luciferensis	0	0	1
Bacillus megaterium	7	1	1
Bacillus mycoides	0	0	1
Bacillus niabensis	0	0	1
Bacillus pumilus	1	6	0
Bacillus simplex	0	0	1
Bacillus sp.	1	0	3
Bacillus subtilis	1	0	1
Brachybacterium faecium	1	0	6
Brachybacterium muris	1	0	1
Brachybacterium nesterenkovii	0	0	2
Brachybacterium sp.	0	0	3
Brevibacillus panacihumi	0	0	1
Brevibacterium casei	8	0	10
Brevibacterium celere	0	1	2
Brevibacterium paucivorans	0	0	2
Brevibacterium ravenspurgense	0	0	3
Candida parapsilosis	3	0	0
Chryseobacterium anthropi	0	0	1
Chryseobacterium hominis	0	0	2
Chryseobacterium sp.	0	0	13
Chryseomicrobium amylolyticum	0	0	1
Citrobacter sp.	1	0	1
Corynebacterium accolens	0	2	11
Corynebacterium afermentans	1	4	25
Corynebacterium argentoratense	0	0	2
Corynebacterium aurimucosum	0	1	13
Corynebacterium coyleae	2	0	18
Corynebacterium durum	2	0	2
Corynebacterium frankenforstense	0	0	1
Corynebacterium glucuronolyticum	0	0	5
Corynebacterium glutamicum	0	0	1
Corynebacterium imitans	0	0	6
Corynebacterium jeikeium	1	2	7
Corynebacterium mastidis	0	0	1
Corynebacterium minutissimum	0	0	1
Corynebacterium mucifaciens	0	0	6
Corynebacterium propinquum	23	1	21
Corynebacterium pseudodiphtheriticum	35	9	3
Corynebacterium resistens	0	0	2
Corynebacterium riegelii	0	0	3
Corynebacterium simulans	0	0	8
Corynebacterium singulare	0	0	1
Corynebacterium sp.	0	0	1
Corynebacterium striatum	0	0	8
Corynebacterium tuberculostearicum	0	0	7

Corynebacterium urealyticum	0	0	2
Dermabacter hominis	0	3	4
Enterobacter aerogenes	0	2	0
Enterococcus faecium	0	0	1
Enterococcus hirae	0	0	2
Gemella haemolysans	1	0	0
Granulicatella adiacens	0	0	1
Klebsiella pneumoniae	0	0	2
Kocuria kristinae	3	1	7
Kocuria marina	0	3	6
Kocuria palustris	1	0	13
Kocuria rhizophila	6	0	15
Kocuria roseus	0	1	0
Kocuria sp.	4	0	3
Kocuria varians	2	0	0
Kytococcus schroeteri	1	0	1
Kytococcus sedentarius	0	0	1
Lactobacillus murinus	1	0	0
Lactobacillus sp.	5	0	1
Lactococcus lactis	0	0	1
Leuconostoc lactis	0	0	1
Microbacterium aurum	4	0	1
Microbacterium lacticum	3	0	3
Microbacterium maritypicum	0	0	3
Microbacterium oxydans	0	0	1
Microbacterium paraoxydans	0	1	3
Microbacterium sp.	0	0	2
Microbacterium testaceum	2	0	12
Micrococcus endophyticus	0	0	2
Micrococcus flavus	10	0	1
Micrococcus luteus	165	70	85
Micrococcus sp.	0	0	1
Moraxella sp.	18	0	5
Moraxella_sg_Branhamella catarrhalis	9	0	0
Moraxella_sg_Moraxella osloensis	73	44	114
Neisseria elongata	1	0	1
Neisseria flavescens	15	2	15
Neisseria macacae	5	0	0
Neisseria mucosa	11	0	0
Neisseria perflava	1	1	1
Neisseria sp.	3	1	1
Neisseria subflava	1	0	0
Not identifiable	3	5	1
Paenibacillus amylolyticus	1	0	0
Paenibacillus ehimensis	0	0	1
Paenibacillus illinoisensis	1	0	0
Paracoccus yeeii	0	2	1
Pseudomonas fulva	0	5	0
Pseudomonas oryzihabitans	1	4	1
Pseudomonas stutzen	0	1	0
Rothia aeria	28	1	4
Rothia amarae	4	0	59
Rothia dentocariosa	12	0	11
Rothia endophytica	1	0	0
Rothia mucilaginosa	12	3	25
Rothia sp.	0	0	4
Rothia terrae	0	0	27
Sphingomonas mucosissima	1	0	0
Sphingomonas paucimobilis	0	0	1
Staphylococcus aureus	23	5	19
Staphylococcus auricularis	0	0	1
Staphylococcus auricularis	0	0	1
Staphylococcus capitis	17	136	224
Staphylococcus caprae	5	0	19
Staphylococcus cohnii	2	0	1
Staphylococcus epidermidis	90	228	118
Staphylococcus haemolyticus	5	11	35
Staphylococcus hominis	79	350	136
Staphylococcus lugdunensis	0	0	4
Staphylococcus pasteuri	3	4	5

Staphylococcus pettenkoferi	0	6	35
Staphylococcus saprophyticus	10	5	7
Staphylococcus schleiferi	0	0	1
Staphylococcus sciuri	0	0	3
Staphylococcus sp.	0	1	2
Staphylococcus warneri	30	9	39
Staphylococcus xylosus	1	0	0
Stenotrophomonas maltophilia	0	0	4
Streptococcus cristatus	0	1	4
Streptococcus gordonii	0	0	7
Streptococcus mitis	18	1	13
Streptococcus mutans	0	0	1
Streptococcus oralis	3	0	5
Streptococcus parasanguinis	9	0	2
Streptococcus peroris	0	0	2
Streptococcus pseudopneumoniae	0	0	1
Streptococcus salivarius	3	0	10
Streptococcus sanguinis	4	0	1
Streptomyces sp.	3	0	1
**total**	842	941	1384
