# Supplementary material for: Skin microbiota analysis-inspired development of novel anti-infectives
Source: Microbiome. 2020 Jun 5;8:85. doi: 10.1186/s40168-020-00866-1 (PMC7275423; doi:10.1186/s40168-020-00866-1)
Supplement: Supplementary file 3 — Additional file 2: Table S2. RNA-Seq results of S. hominis S34-1 wild-type versus S34-1Δ comparison [file 40168_2020_866_MOESM2_ESM.pdf]

Additional Table 2. RNA-Seq results of *S. hominis* S34-1wild-type versus S34-1Δ comparison

Gene ID	S34-1 vs. ΔS34-1 Fold change	Annotation	P value
Down-regulated in mutant			
plasmid1_gene0006	0.1974148	MerR family transcriptional regulator [Lachnoclostridium]	1.97116E-84
plasmid1_gene0013	0.4659825	hypothetical protein [Bacillus Hypothetical Cytosolic Protein [Bacillus cereus BDRD-Cer4]	5.98701E-18
plasmid1_gene0017	0	thiocillin [Bacillus cereus]	6.8819E-62
plasmid1_gene0022	0.3853318	replication protein [Staphylococcus warneri]	9.90409E-26
plasmid1_gene0023	0.2518031	glyoxalase [Staphylococcus glyoxalase family protein [Staphylococcus hominis SK119]	2.06038E-13
gene0016	0.4995521	adenylosuccinate synthetase [Staphylococcus hominis]	1.90587E-10
gene0174	0.3635099	hypothetical protein [Salimicrobium sp. MJ3]	0.044520716
gene0283	0.310019	hypothetical protein [Staphylococcus hominis]	0.00158768
gene0315	0.3165306	phosphoadenosine phosphosulfate reductase [Staphylococcus hominis]	5.537E-55
gene0316	0.4732922	sulfite reductase subunit alpha [Staphylococcus hominis]	5.63013E-28
gene0317	0.4340149	sulfite reductase [Staphylococcus hominis]	9.20145E-35
gene0318	0.4745097	uroporphyrin-III C-methyltransferase [Staphylococcus hominis]	1.33711E-24
gene0319	0.3992826	precorrin-2 dehydrogenase [Staphylococcus hominis]	5.8159E-36
gene0320	0.3365521	membrane protein [Staphylococcus hominis]	6.80074E-47
gene0321	0.3148598	sulfate adenylyltransferase [Staphylococcus sp. MDS7B]	1.97457E-51
gene0322	0.4232978	adenylylsulfate kinase [Staphylococcus hominis]	4.73001E-20
gene0323	0.3435355	Secretory antigen precursor SsaA [Staphylococcus haemolyticus]	5.42748E-38
gene0362	0.457624	transglycosylase [Staphylococcus hominis]	2.36212E-16
gene0459	0.1974661	antiholin antiholin-like protein LrgB [Staphylococcus hominis SK119]	1.31418E-63
gene0460	0.2689859	murein hydrolase regulator LrgA [Staphylococcus hominis]	5.45027E-06
gene0585	0.4100433	hypothetical protein [Staphylococcus hominis]	3.60897E-14
gene0798	0.4052194	threonine dehydratase [Staphylococcus sp. MDS7B]	4.7018E-10
gene0799	0.2999272	isopropylmalate isomerase [Staphylococcus sp. MDS7B]	0.005190309
gene0800	0.3581454	3-isopropylmalate dehydratase large subunit [Staphylococcus sp. M0480]	4.6616E-22
gene0801	0.3364761	3-isopropylmalate dehydrogenase [Staphylococcus hominis]	2.1155E-09
gene0803	0.4749364	ketol-acid reductoisomerase [Staphylococcus]	1.48717E-07
gene0865	0.4159535	hypothetical protein [Staphylococcus]	0.001655029
gene0880	0.4293661	hypothetical protein [Staphylococcus]	0.040796675
gene1164	0.4882841	DNA repair protein RecO [Staphylococcus hominis]	2.7669E-06
gene1198	0.3286667	hypothetical protein [Staphylococcus]	0.003210093
gene1388	0.07568076	hypothetical protein [Staphylococcus hominis]	1.33371E-05
gene1427	0.4999374	30S ribosomal protein S15 [Staphylococcus hominis]	1.52901E-10
gene1498	0.4836645	carbamoyl phosphate synthase small subunit [Staphylococcus]	4.0337E-26
gene1641	0.1983068	hypothetical protein [Staphylococcus hominis]	0.007495549
gene1647	0.3457724	hypothetical protein [Staphylococcus hominis]	1.10924E-05
gene1670	0.4924082	peptide ABC transporter substrate-binding protein [Staphylococcus hominis]	9.4584E-11
gene1925	0.3462109	MerR family transcriptional regulator [Staphylococcus hominis]	7.0314E-17
gene1970	0.1643757	-	0.001583033
gene2097	0.4948841	methionine ABC transporter substrate-binding protein [Staphylococcus]	5.24376E-09
gene2098	0.4699187	methionine ABC transporter permease [Staphylococcus hominis]	8.82595E-07
gene2102	0.4515412	cysteine synthase [Staphylococcus hominis]	2.62215E-26
Up-regulated in mutant			

gene0163	3.323146	hypothetical protein [Staphylococcus hominis]	2.80494E-68
gene0215	2.249664	diacetyl reductase [(S)-acetoin forming]	1.13075E-38
gene0347	2.097634	alpha-acetolactate decarboxylase [Staphylococcus hominis]	9.5416E-06
gene0369	2.134515	short-chain dehydrogenase [Staphylococcus sp. MDS7B]	1.80545E-32
gene0412	2.040101	acetyltransferase [Staphylococcus hominis]	1.54116E-10
gene0419	2.030836	MoeB [Staphylococcus sp. MDS7B]	1.33826E-05
gene0437	2.889369	hypothetical protein [Staphylococcus]	4.85642E-21
gene0455	2.365682	glycine/betaine ABC transporter ATP-binding protein [Staphylococcus sp. MDS7B]	6.37696E-13
gene0456	2.321234	choline ABC transporter permease [Staphylococcus hominis]	1.30728E-12
gene0457	2.677133	choline ABC transporter permease [Staphylococcus]	2.88621E-32
gene0458	2.970472	glycine/betaine ABC transporter substrate-binding protein [Staphylococcus hominis]	4.36403E-50
gene0474	2.029366	cell wall biosynthesis protein ScdA [Staphylococcus hominis]	2.61619E-06
gene1592	2.339765	cytochrome D ubiquinol oxidase subunit II [Staphylococcus]	1.88039E-29
gene1919	2.167976	hypothetical protein [Staphylococcus hominis]	2.56687E-17
gene2162	2.240674	hypothetical protein [Staphylococcus sp. MDS7B]	1.48568E-13
